# Altered enhancer transcription underlies Huntington’s disease striatal transcriptional signature

**DOI:** 10.1038/srep42875

**Published:** 2017-02-22

**Authors:** Stéphanie Le Gras, Céline Keime, Anne Anthony, Caroline Lotz, Lucie De Longprez, Emmanuel Brouillet, Jean-Christophe Cassel, Anne-Laurence Boutillier, Karine Merienne

**Affiliations:** 1 GenomeEast Platform, Institut de Génétique et de Biologie Moléculaire et Cellulaire (IGBMC), CNRS/INSERM/University of Strasbourg—UMR 7104, 1 rue Laurent Fries, 67404 Illkirch, France; 2 University of Strasbourg, Laboratory of Cognitive and Adaptive Neurosciences (LNCA), 12 rue Goethe, 67000 Strasbourg, France; 3 CNRS, LNCA UMR 7364, 12 rue Goethe, 67000 Strasbourg, France; 4 Commissariat à l’Energie Atomique (CEA), Département de Recherches Fondamentales (DRF), Institut d’Imagerie Biomédicale (I2BM), Molecular Imaging Research Center (MIRCen), F-92260 Fontenay-aux-Roses, France; 5 Centre National de la Recherche Scientifique (CNRS), Université Paris-Sud, Université Paris-Saclay, UMR 9199, Neurodegenerative Diseases Laboratory, F-92260 Fontenay-aux-Roses, France

## Abstract

Epigenetic and transcriptional alterations are both implicated in Huntington’s disease (HD), a progressive neurodegenerative disease resulting in degeneration of striatal neurons in the brain. However, how impaired epigenetic regulation leads to transcriptional dysregulation in HD is unclear. Here, we investigated enhancer RNAs (eRNAs), a class of long non-coding RNAs transcribed from active enhancers. We found that eRNAs are expressed from many enhancers of mouse striatum and showed that a subset of those eRNAs are deregulated in HD *vs* control mouse striatum. Enhancer regions producing eRNAs decreased in HD mouse striatum were associated with genes involved in striatal neuron identity. Consistently, they were enriched in striatal super-enhancers. Moreover, decreased eRNA expression in HD mouse striatum correlated with down-regulation of associated genes. Additionally, a significant number of RNA Polymerase II (RNAPII) binding sites were lost within enhancers associated with decreased eRNAs in HD *vs* control mouse striatum. Together, this indicates that loss of RNAPII at HD mouse enhancers contributes to reduced transcription of eRNAs, resulting in down-regulation of target genes. Thus, our data support the view that eRNA dysregulation in HD striatum is a key mechanism leading to altered transcription of striatal neuron identity genes, through reduced recruitment of RNAPII at super-enhancers.

Huntington’s disease (HD), a progressive neurodegenerative disease affecting primarily medium spiny neurons of the striatum, leads to cognitive, motor and mood impairments. As for several neurodegenerative diseases, including Alzheimer’s and Parkinson’s diseases, neuronal dysfunction in HD correlates with epigenetic changes, particularly changes in histone modifications^1^,^2^,^3^,^4^,^5^,^6^,^7^. However, the mechanisms underlying epigenetic alterations in HD striatal neurons and their consequences on HD pathogenesis remain unclear.

Transcriptional dysregulation in HD is tissue-dependent and most extensive in the striatum^8^,^9^,^10^. Specifically, HD striatum displays a “neuronal” transcriptional signature^1^,^11^, characterized by down-regulation of many genes implicated in biological processes linked to neuronal activity, such as neuronal transmission and excitability, synaptic plasticity and learning^2^,^4^,^11^. Noticeably, down-regulated genes in HD striatum are enriched in striatal markers, *i.e*. genes essential to the function and identity of striatal neurons^2^,^10^,^12^,^13^. Typically, these genes, which are highly expressed in the striatum, comprise neuronal receptors, ion channels and signaling factors (*e.g*. DRD1, DRD2, KCNJ4, RGS9, DARPP32) required for proper regulation of striatal neuron activity. It is considered that transcriptional down-regulation underlies dysfunction of striatal neurons, preceding neuronal death, and may thus be a key mechanism of HD striatal pathogenesis^1^,^8^,^14^.

Using genome-wide scale approaches, we previously showed that the enhancer mark H3K27 acetylation (H3K27ac) was selectively decreased at super-enhancers, a category of broad enhancers regulating cell type-specific identity genes, and this event correlated with decreased expression of super-enhancer target genes^2^. This suggests that altered super-enhancer activity contributes to repression of neuronal genes in HD mouse striatum and to the establishment of HD “neuronal” transcriptional signature.

Regulation of enhancer activity involves enhancer transcription, *i.e*. transcription of long non-coding RNAs, called enhancer RNAs (eRNAs)^15^,^16^. eRNAs, which are transcribed from active, tissue-specific enhancers, are generally positively correlated with transcription of their target genes^15^,^16^,^17^,^18^,^19^. Recent studies provide evidence for a causative role for eRNAs in regulating target genes transcription^18^. Specifically, eRNAs precede and activate transcription of target genes, influencing chromatin looping between enhancer and promoter, and modulating RNA polymerase II (RNAPII) dynamics^18^,^20^,^21^.

Here we show that dysregulation of enhancer transcription is extensive in HD mouse striatum. Our data further indicate that eRNA dysregulation in HD mouse striatum results from altered recruitment of RNAPII at super-enhancers and underlies down-regulation of striatal marker genes. Thus, we provide new insights into the epigenetic mechanism underlying repression of striatum-specific identity genes.

## Results

### Differential expression of eRNAs in the striatum of HD R6/1 mice

**Figure 1:**
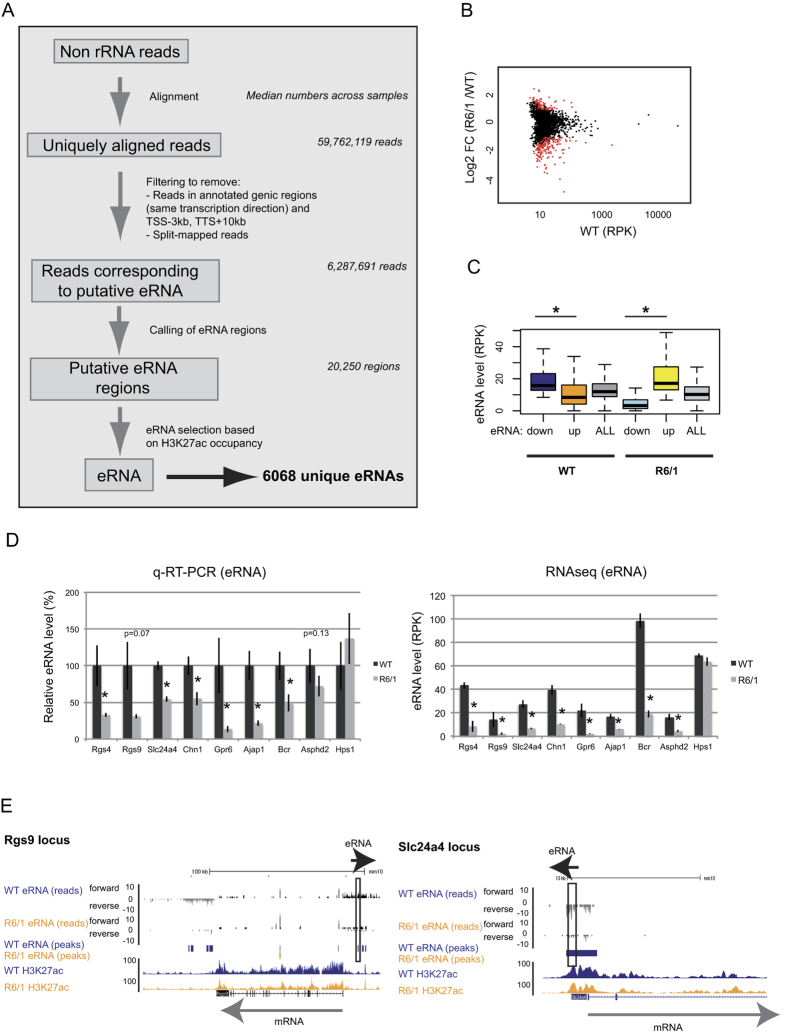
Identification, validation and global features of eRNAs in WT and HD R6/1 mouse striatum. (**A**) Workflow showing the sequential steps to identify eRNAs using RNAseq and H3K27ac ChIPseq data generated in striatum of WT and R6/1 mice. 6068 unique eRNAs were identified. (**B**) Scatter plot analysis of eRNAs, showing in red up- and down-regulated eRNAs in R6/1 vs WT mouse striatum. (**C**) Boxplot representation showing that WT levels of down-regulated and up-regulated eRNAs in R6/1 vs WT striatum are high and low, respectively. The situation is opposite when considering R6/1 samples. *P < 0.05 (Wilcoxon test). (**D**) (Left) Validation of eRNAs associated with *Rgs4, Rgs9, Slc24a4, Chn1, Gpr6, Ajap1, Bcr, Asphd2* and *Hps1* using q-RT-PCR. Error bars, sem; *P < 0.05 (Student’s t-test). (Right) Analysis of RNAseq data showing that eRNA retrieved from regions tested by q-RT-PCR (at *Rgs4, Rgs9, Scl24a4, Chn1, Gpr6, Ajap1, Bcr and Asphd2* loci) are decreased, while eRNA associated with *Hps1* is unchanged. Error bars, sem; *P < 0.05 (Adjusted p-values, see methods). (**E**) Genome browser representation showing *Rgs9* and *Slc24a4* loci, including eRNA reads (before H3K27ac filtering), eRNA peaks (after H3K27ac filtering) and H3K27ac signals in WT and R6/1 striatum. Black boxes delimitate eRNA regions that were validated using q-RT-PCR. Black and grey arrows show the direction of expression of eRNA and mRNA, respectively (*Rgs4* and *Hps1* loci are shown in Fig. S1).

To explore the hypothesis that alteration of eRNA transcription might be a component of HD pathogenesis, we assessed eRNAs at genome-wide scale, analyzing non-coding RNAs from RNA sequencing (RNAseq) data previously generated in the striatum of control and HD R6/1 transgenic mice^2^. A strand-specific total RNA sequencing protocol was used to generate sequencing reads, thereby allowing analysis of long non-coding RNAs (see Methods). To identify eRNAs, *i.e*. non-coding RNAs synthesized from enhancers, we first excluded signals within genic regions, defined as the interval starting 3 kb upstream of the transcription start site and ending 10 kb downstream of the transcription termination site, since they might result from polymerase read-through of genic transcripts (ref. 19 and Methods). Second, we filtered RNA signals resulting from enhancer regions using H3K27ac ChIP-seq data, generated from the striatum of control (WT) and HD R6/1 mice^2^. As a result, a total of 6068 eRNAs were selected based on H3K27ac occupancy (Fig. 1A). Analysis of differentially expressed eRNAs between R6/1 and WT striata was performed, as well as eRNA annotation, which provided gene-eRNA associations (see Methods). 677 and 335 eRNAs were found decreased and increased, respectively (Fig. 1B and Table S1). Noticeably, down-regulated eRNAs were globally expressed at higher levels than up-regulated eRNAs in WT mice (Fig. 1C). Decreased expression of selective eRNAs, including eRNAs in neighborhood of *Rgs4, Rgs9, Slc24a4, Chn1, Gpr6, Ajap1, Bcr and Asphd2* genes, was confirmed by q-RT-PCR (Fig. 1D,E and S1A). Expression of *Hps1*-associated eRNA, which was unchanged between R6/1 and WT, was used as a negative control (Fig. 1D,E and S1A). mRNAs transcribed from *Rgs4, Rgs9, Slc24a4, Chn1, Gpr6, Ajap1, Bcr and Asphd2* were also decreased, in contrast to *Hps1* mRNA (Fig. S1B), suggesting a link between eRNA and mRNA deregulation in R6/1 mouse striatum. To evaluate the degree of conservation of the mechanism, we analyzed another HD mouse model widely used in the field, the Q140 knockin model, expressing full-length mutant Htt. These mice display progressive transcriptional dysregulation, particularly in the striatum^10^. *Rgs4, Rgs9, Slc24a4, Chn1, Gpr6, Ajap1, Bcr and Asphd2* mRNAs were also decreased in the striatum of 12 month-old Q140 mice (ref. 10 and S1B). eRNAs associated with these genes were significantly decreased or showed a tendency to the decrease, except *Slc24a4*-associated eRNA (Fig. S1C), suggesting that eRNA dysregulation in HD striatum is a general mechanism.

### Target genes of decreased eRNAs in R6/1 striatum are enriched in neuronal function genes

**Figure 2:**
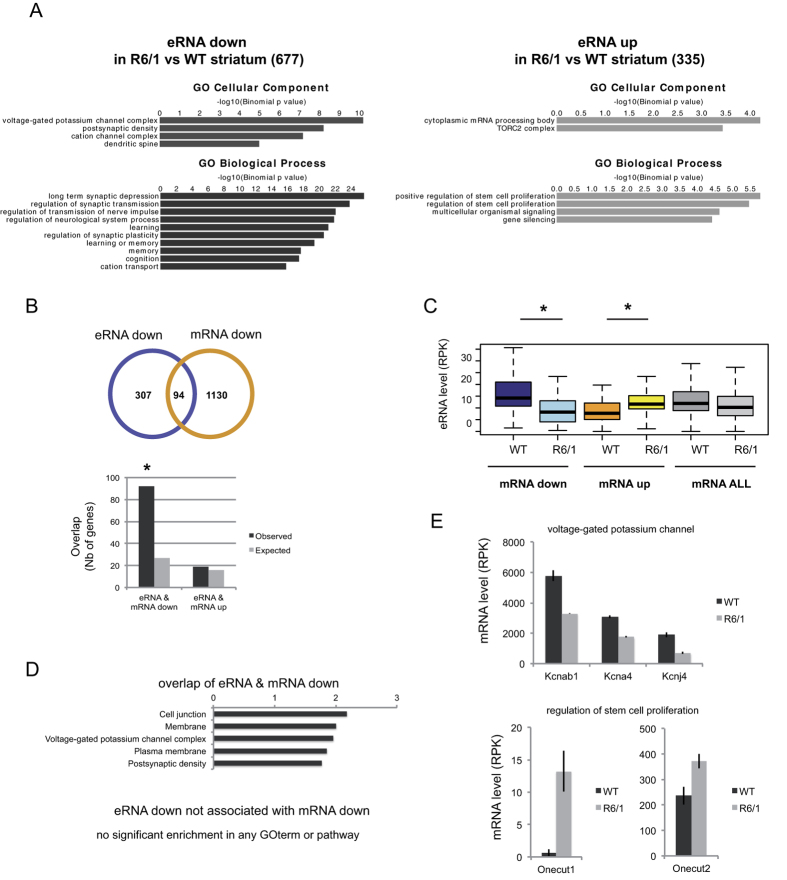
Target genes of decreased eRNAs in R6/1 striatum are involved in neuronal activity and are enriched in down-regulated genes. (**A**) Functional enrichment analysis of down- and up-regulated eRNAs in R6/1 vs WT striatum showing that down-regulated eRNAs (i.e. 677 down eRNAs) are strongly enriched in cellular components and biological processes linked to neuronal function, and thus display a clear neuronal signature. In contrast, up-regulated eRNAs (i.e. 335 up eRNAs) present stem cell proliferation and gene silencing signatures. (**B**) (Up) Overlap between target genes of decreased eRNAs and down-regulated genes in R6/1 vs WT striatum. (Down) Target genes of decreased eRNAs in R6/1 vs WT striatum are significantly enriched in down-regulated genes, whereas target genes of increased eRNAs in R6/1 vs WT striatum are not enriched in up-regulated genes. Observed numbers are compared to expected numbers; *P < 10^−2^; Chi-square test. (**C**) Boxplot representation showing eRNA levels associated with down-regulated, up-regulated or all genes in R6/1 and WT striatum. *P < 0.05 (Wilcoxon test). (**D**) Functional enrichment analysis of eRNAs that were both decreased in R6/1 vs WT striatum and associated with down-regulated target gene. Decreased eRNAs associated with down-regulated genes in R6/1 vs WT striatum display a neuronal signature, noticeably a voltage-gated potassium channel signature, whereas decreased eRNAs that do not associate with down-regulated genes do not present any specific functional signature. (**E**) Target genes of decreased and increased eRNAs contributing, respectively, to voltage-gated potassium channel (*e.g. Kcnab1, Kcna4, Kcnj4*) and stem cell (*e.g. Onecut1* and *Onecut2*) signatures. mRNA levels calculated from RNAseq data are shown. Error bars, sem; (Adjusted p-values < 0.05, see methods).

We investigated whether down-regulated eRNAs in R6/1 striatum were enriched in genes implicated in specific functions. Gene ontology analysis (GO) using GREAT^22^ showed that enhancer regions involved in decreased eRNAs in R6/1 striatum were strongly associated with genes enriched in biological processes linked to neuronal activity, including neuronal transmission, synaptic plasticity and learning and memory (Fig. 2A). In contrast, regions involved in increased eRNAs were close to genes enriched in biological processes related to stem cell proliferation (Fig. 2A). Thus, down- and up-regulated eRNAs in R6/1 striatum associate with genes that display neuronal and developmental signatures, respectively.

### Target genes of decreased eRNAs in R6/1 striatum are enriched in down-regulated genes

Down-regulated genes in R6/1 striatum also present a strong neuronal signature^2^. Since eRNAs positively regulate their target genes, this suggests that decreased eRNAs might modulate expression of genes down-regulated in R6/1 striatum. Integrated analysis showed that target genes of enhancers associated with decreased eRNAs in R6/1 striatum were enriched in down-regulated genes (Fig. 2B). Moreover, levels of eRNAs in the neighborhood of decreased mRNA in R6/1 striatum were globally reduced (Fig. 2C). As expected, the subset of down-regulated genes associated with decreased eRNAs in R6/1 striatum displayed a clear neuronal signature (Fig. 2D). Interestingly, they were enriched in genes controlling neuronal excitability, including genes coding for voltage-gated potassium channels such as *Knca4, Kcnab1* and *Kcnj4* (Fig. 2E). In contrast, target genes of increased eRNAs were not enriched in up-regulated genes in R6/1 striatum (Fig. 2B). However, eRNAs associated with up-regulated genes in R6/1 *vs* WT striatum were globally increased (Fig. 2C), and these genes included developmental genes such as *Onecut1* and *Onecut2*, expressed in neural stem cells (Fig. 2E and ref. 23). Together, these results suggest that eRNA down-regulation has a broader influence on gene expression than eRNA up-regulation in R6/1 striatum, with biological impact of decreased eRNAs in R6/1 striatum affecting neuronal activity, including neuronal excitability, and that of increased eRNAs influencing neuronal fate. These two effects of eRNA dysregulation might synergistically contribute to loss of neuronal differentiated state of HD striatal neurons.

### Altered transcribed enhancers in R6/1 striatum are enriched in super-enhancers

**Figure 3:**
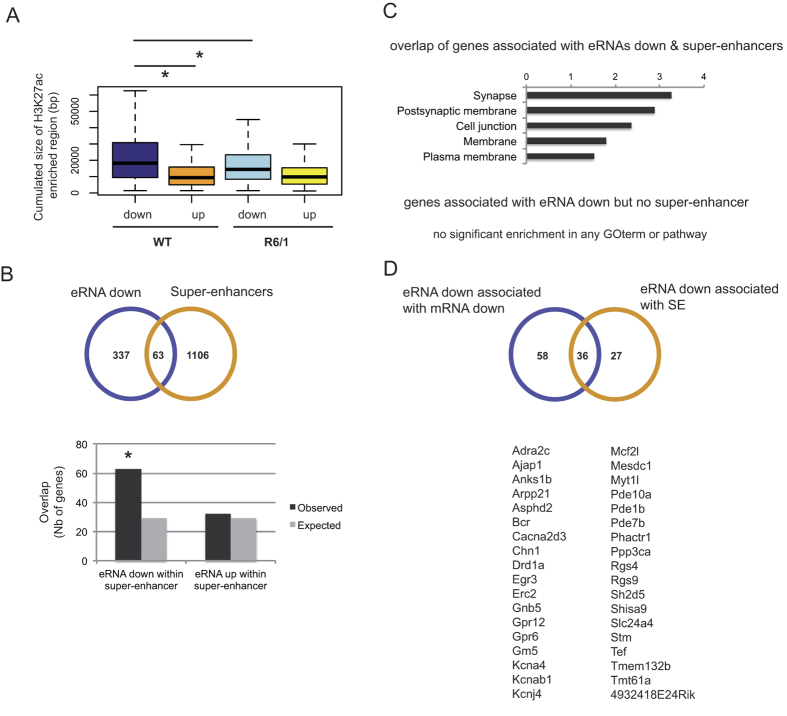
Enhancers leading to decreased eRNAs are enriched in super-enhancers in R6/1 striatum. (**A**) Enhancer size of decreased eRNAs (down) in R6/1 vs WT striatum is broad and reduced in R6/1 striatum, compared to enhancer size of increased eRNAs (up). *P < 0.05 (Wilcoxon test). (**B**) (Up) Overlap of target genes of decreased eRNAs and genes regulated by a super-enhancer. (Down) Decreased eRNAs are enriched in eRNAs produced from super-enhancers, in contrast to increased eRNAs. Observed numbers are compared to expected numbers; *P < 10^−2^; Chi-square test. (**C**) Functional enrichment analysis of decreased eRNAs in R6/1 striatum that are produced from a super-enhancer. Target genes of these decreased eRNAs display a neuronal signature, in contrast to target genes of decreased eRNAs not produced from a super-enhancer, which do not display any functional signature. (**D**) (Up) Overlap of target genes of decreased eRNAs that are both down-regulated in R6/1 striatum and regulated by a super-enhancer (SE). (Down) Gene names of this sub-list are presented.

Target genes of super-enhancers, a category of broad enhancers regulating cell type-specific identity genes, are preferentially down-regulated in R6/1 striatum^2^. We therefore hypothesized that decreased eRNAs in R6/1 striatum were transcribed from super-enhancers. Integrated analysis of eRNA and H3K27ac ChIP-seq data on R6/1 and WT striatum supported this hypothesis, since H3K27ac-enriched regions associated with decreased eRNAs were broader in comparison with those associated with increased eRNAs (Fig. 3A). Consistently, enhancers associated with down-regulated eRNAs in R6/1 striatum were enriched in super-enhancers, in contrast to up-regulated eRNAs (Fig. 3B). In addition, decreased eRNAs produced from super-enhancers displayed a strong neuronal signature, whereas those produced from typical enhancers did not present any specific functional signature (Fig. 3C). This shows that neuronal signature of down-regulated eRNAs is essentially contributed by transcribed super-enhancers. We also crossed the list of decreased eRNA-associated genes regulated by a super-enhancer with the list of decreased eRNA-associated genes down-regulated in R6/1 striatum. Both lists were largely overlapping (Fig. 3D), showing that most down-regulated target genes associating with reduced eRNAs in R6/1 *vs* WT striatum are under the control of a super-enhancer. Genes within the resulting sub-list (36 genes) contained striatal markers (*e.g. Drd1, Rgs9*), including striatal voltage-gated potassium channels (*e.g. Kcnj4*/*Kir2.3*, ref. 24) (Fig. 3D). Finally, the size of H3K27ac enriched regions at enhancers associated with decreased eRNAs in R6/1 striatum were reduced in R6/1 when compared to WT mice (Fig. 3A), suggesting a link between reduction of H3K27ac-enriched regions and eRNA transcription in R6/1 striatum. Together, these data indicate that altered super-enhancer transcription in HD mouse striatum contributes to down-regulation of striatal identity genes.

### RNAPII binding sites are lost at enhancers associated with decreased eRNAs in R6/1 striatum

**Figure 4:**
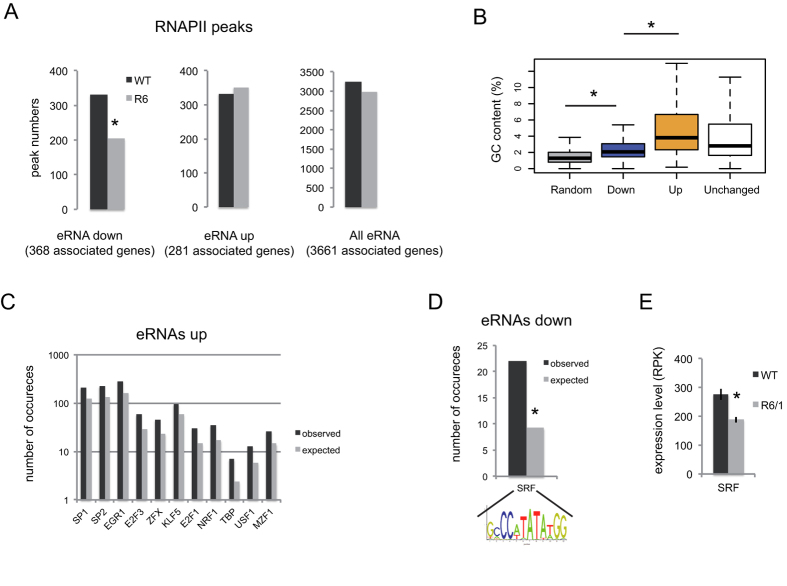
Enhancers leading to decreased eRNAs in R6/1 vs WT striatum present reduced RNAPII signals in R6/1 striatum and are enriched in SRF binding sites. (**A**) Graphs showing the numbers of RNAPII peaks in R6/1 and WT striatum found at enhancers leading to decreased and increased eRNAs in R6/1 vs WT striatum. RNAPII peaks numbers, when considering all eRNAs, are also shown (**B)** GC content at transcribed enhancers is elevated, as compared to randomly chosen genomic sequences (Random), but less so at enhancers leading to decreased eRNAs (Down) in R6/1 striatum, when compared to enhancers leading to increased eRNAs (Up) or unchanged eRNAs (Unchanged) in R6/1 vs WT striatum. (**C**) DNA motif analysis showing that increased eRNAs in R6/1 vs WT striatum are enriched in several DNA motifs. Observed numbers of occurrences are compared to expected numbers. Only significantly enriched DNA motifs are shown (see methods). (**D**) DNA motif analysis showing that decreased eRNAs in R6/1 vs WT striatum are only enriched in the SRF DNA motif. Observed numbers of occurrences are compared to expected numbers. (**E**) Expression level of SRF in R6/1 vs WT striatum is shown (RNAseq data).

RNAPII is enriched at start sites of transcribed enhancers^16^,^19^,^25^. To investigate whether RNAPII signal varied between WT and R6/1 samples, we integrated RNAPII ChIPseq data previously generated^2^ to the analysis, filtering eRNAs containing RNAPII peaks. Out of 6068 eRNAs, 3248 were retrieved in WT mice following this filtering. A similar number (2990) were identified from R6/1 mice (Fig. 4A). Thus, a substantial proportion (≈1/2) of eRNAs were associated with a RNAPII peak and the numbers of eRNAs with RNAPII peaks were similar between WT and R6/1 samples, though a slight decrease (8%) was observed in R6/1 *vs* WT striatum. 331 eRNAs with RNAPII peaks were decreased in R6/1 striatum when compared to WT. Out of these 331 eRNAs, 127 lost RNAPII peaks in the R6/1 condition, which corresponded to a 38% loss. Thus, RNAPII peaks were dramatically reduced at R6/1 enhancers associated with decreased eRNAs (Fig. 4A), suggesting that loss of RNAPII contributes to decreased transcription of eRNA in HD mouse striatum. In contrast, 351 eRNAs were found increased in R6/1 *vs* WT striatum, of which only 18 gained RNAPII peaks in R6/1 mice (*e.g*. 5%; Fig. 4A), suggesting that RNAPII gain may not be a major mechanism governing eRNA upregulation in R6/1 striatum.

### Enhancers associated with deregulated eRNAs in R6/1 striatum are enriched in selective DNA motifs

We then asked whether deregulated eRNAs in R6/1 striatum were enriched in binding sites for transcriptional regulators. Remarkably, transcribed enhancer sequences, whether they led to deregulated eRNAs or not, were enriched in GC content (Fig. 4B). This was particularly true for up-regulated eRNAs (Fig. 4B), which were also enriched in GC-rich DNA motifs (Fig. 4C and S1D). Within the list of DNA binding sites enriched in up-regulated eRNAs, none were recognized by transcriptional activators substantially up-regulated or transcriptional repressors down-regulated in the striatum of R6/1 mice^2^. However, *Klf5* and *Usf1* were slightly but significantly up-regulated in R6/1 striatum (adjusted p-value is 0.03 in both cases, ref. 2). Usf1 acts as a chromatin insulator element, promoting gene activation through inhibition of polycomb complex activity^26^, whereas Klf5 is a developmental transcription factor involved in maintenance of embryonic stem cells undifferentiated state^27^,^28^. These DNA motif signatures are consistent with the functional signature of up-regulated eRNAs in R6/1 striatum, characterized by biological processes linked to both gene silencing and regulation of stem cell proliferation (Fig. 2A).

In contrast to increased eRNAs in R6/1 striatum, decreased eRNAs were exclusively enriched in a DNA motif recognized by SRF (Fig. 4D), a transcription factor that recruits RNAPII to transcription sites. Additionally, *Srf* expression, which is substantial in mouse striatum, is significantly decreased in HD mouse striatum (Fig. 4E). Since SRF is a key player of neuronal plasticity^29^, reduced *Srf* expression in HD striatum might contribute to decreased transcription at striatal enhancers, through impaired recruitment of RNAPII, which might in turn lead to down-regulation of striatal enhancer target genes.

## Discussion

In this study, we have investigated enhancer transcription in normal and Huntington’s disease mouse striatum. We show that eRNAs are widely expressed from striatal enhancers and many eRNAs are decreased in R6/1 *vs* WT mouse striatum. These decreased eRNAs in R6/1 striatum display a strong neuronal signature: their target genes are highly enriched in biological processes linked to synaptic plasticity, neuronal transmission and learning. This result is consistent with the fact that decreased eRNAs in R6/1 striatum are preferentially expressed from super-enhancers, which control genes that define cell type-specific identity and function. Our data also show that transcribed enhancers resulting in decreased eRNAs in R6/1 striatum are selectively enriched in a DNA motif recognized by SRF, a transcription factor altered in R6/1 striatum, display loss of RNAPII signals, and associate with genes that are down-regulated in R6/1 striatum. Together, these data indicate that repression of neuronal genes in HD striatum involves an epigenetic mechanism, eRNA dysregulation, originating from altered recruitment of RNAPII at super-enhancers, possibly due to reduced SRF levels.

Down-regulated genes in HD mouse striatum are preferentially regulated by super-enhancers^2^. As a result, they display a strong neuronal signature^2^,^4^,^9^,^10^. In this study, we showed that transcription at substantial numbers of super-enhancers was decreased in R6/1 striatum (Fig. 3). Target genes of super-enhancers showing decreased eRNAs were down-regulated in R6/1 striatum (Fig. 2). Since enhancer transcription is a marker of enhancer activity^16^ and eRNAs positively regulate transcription of their target genes^18^, our results uncover that altered super-enhancer activity broadly contributes to down-regulation of markers of striatal identity in HD mice.

In contrast, increased (super-) enhancer activity had no broad impact on gene up-regulation in HD striatum, since up-regulated eRNAs in R6/1 striatum poorly overlapped with super-enhancers and their target genes were not enriched in up-regulated genes (Fig. 2B). However, our results showed that up-regulation of the developmental genes *Onecut1* and *Onecut2*, expressed in neural stem cells^23^, correlated with augmented transcription of associated eRNAs (Fig. 2E). This suggests that selected increased eRNAs contribute to striatal neuron dedifferentiation. Thus, increased and decreased transcription at enhancers might both participate to loss of differentiated state of striatal neurons in response to the HD mutation, through re-activation of genes expressed in immature neurons and repression of genes defining striatal neuron identity, respectively.

Using a cellular model of inflammation, Hah *et al*. reported extensive transcription within super-enhancers^19^. They also showed that super-enhancer transcription was required for the regulation of target genes involved in innate immunity. Our results showed that neuronal super-enhancer transcription was also extensive, which suggests that super-enhancer transcription is a general mechanism critical to the regulation of genes that determine cell type-specific identity and function.

We analyzed eRNA transcription in basal conditions – as opposed to stimulus-induced conditions – and found that many eRNAs (6068) were transcribed from mouse striatal enhancers. This result is consistent with previous study, which identified 8990 and 7779 eRNAs in mouse cortex and cerebellum, respectively^30^. Thus, many enhancers are constitutively active in a mouse brain tissue and our results indicate that repression of neuronal genes in HD striatum predominantly results from altered activity of constitutively active enhancers.

Using primary cortical cultures, Kim *et al*. previously identified neuronal stimulus-responsive enhancers using a genome-wide approach^16^. The epigenetic signature of these activity-regulated enhancers was characterized by increased H3K27ac signals and augmented eRNA transcription^16^,^17^. Our results showing that reduced eRNAs transcription correlates with decreased H3K27ac signals in R6/1 striatum (Fig. 3A) further support the idea of an interaction between mechanisms regulating enhancer transcription and H3K27 acetylation.

The transcription factor FOS was enriched at neuronal stimulus-responsive enhancers^17^. However, it is not likely to be the case for constitutively active enhancers, since basal expression of *Fos* is low in neurons. Remarkably, enhancers that associated with reduced eRNA transcription in HD mouse striatum were enriched in a DNA motif recognized by another transcription factor, SRF (Fig. 4D). SRF is a key regulator of synaptic plasticity and this function of SRF includes mechanisms that modulate constitutively expressed genes^31^. Particularly, basal gene expression changes resulting from inactivation of the *Srf* gene in the adult forebrain accounted for altered synaptic plasticity, specifically long term depression (LTD)^31^. Noticeably, it has been reported that synaptic plasticity, including LTD, is impaired in HD mice^32^. Moreover, *Srf* transcription is reduced in R6/1 striatum (Fig. 4E). Whether altered SRF regulation in HD mouse striatum affects constitutively active enhancers, including super-enhancers, thereby contributing to altered expression of synaptic plasticity genes, is an intriguing hypothesis. This possibility would be consistent with functional enrichment analysis showing that down-regulated eRNAs in R6/1 striatum were enriched in GO terms such as “regulation of synaptic plasticity”, “long term depression” (LTD) and “learning” (Fig. 2A).

Voltage-gated potassium channels were enriched in the subsets of down-regulated genes associated with reduced enhancer transcription (Fig. 2D). Specific regulation of potassium currents is required to unique electrophysiological properties of striatal neurons, including maintenance of the hyperpolarized state^24^,^33^,^34^. Decreased expression of voltage-gated potassium channels in HD mice, which results in altered excitability properties of striatal neurons, due to depolarized resting state membrane potentials, was suggested to partially account for preferential vulnerability of striatal medium spiny neurons in HD^24^. Our data showing that altered transcribed enhancers in HD mouse striatum display a voltage-gated potassium channel signature might indicate that eRNA dysregulation underlies increased striatal vulnerability in HD.

We showed that transcribed enhancers display high GC content (Fig. 4B), which suggests that enhancers are subject to regulation by DNA methylation. Strikingly, decreased eRNAs in R6/1 *vs* WT striatum were less GC-rich, in comparison with up-regulated eRNAs or with unchanged eRNAs. This is consistent with results showing that super-enhancers are hypo-methylated, which might facilitate a chromatin state permissive to transcription^35^,^36^.

In conclusion, we propose that targeting striatal enhancers in an attempt to improve enhancer transcription might prevent repression of neuronal genes in HD. The development of therapeutic strategies permitting to target enhancers may thus represent a future challenge.

## Materials and Methods

### Animals

Hemizygous R6/1 (≈150 CAGs)^37^ and Q140 knockin mice from the Jackson Laboratory were maintained on mixed CBAxC57BL/6 and C57BL/6 genetic backgrounds, respectively. The experimental protocol followed the European directive (Directive 2010/63/UE) and received French governmental authorizations. Mice were housed in a temperature-controlled room maintained on a 12 hours light/dark cycle. Food and water were available ad libitum. All animal studies were conducted according to the French regulation (EU Directive 2010/63/EU – French Act Rural Code R 214-87 to 131). The animal facility was approved by veterinarian inspectors: authorizations n° E67-482-13 for Laboratory of Cognitive and Adaptive Neurosciences (i.e. LNCA) and n° A 92-032-02 for Commissariat à l’Energie Atomique (i.e. CEA) and complies with Standards for Humane Care and Use of Laboratory Animals of the Office of Laboratory Animal Welfare (OLAW). All procedures received approval from French Ministry of Higher Education and Research (authorizations n° 2015042011568820v3/APAFIS#504 for R6/1 line/LNCA and n° 2015060417243726vl/APAFIS#770 for Q140 line/CEA). People involved in animal care, killing and tissue preparation have official expertise. R6/1 and Q140 mice were killed at 30 weeks and 12 months, respectively, and their striata were rapidly dissected, snap frozen and stored at −80 °C. The methods regarding animal use were carried out in accordance with the relevant guidelines and regulations.

### RNA extraction and qRT-PCR

Each sample was prepared from single striatum of R6/1 and WT littermate mice (for R6/1 *vs* WT comparisons) or from single striatum of Q140 and WT littermate mice (for Q140 *vs* WT comparisons). Biological replicates (4 to 6) were performed for each group. Tissues were finely cut with a razor blade and total RNA was extracted using TRIzol reagent (Invitrogen). An additional DNAse treatment (Euromedex) was added before RNA purification by phenol/chloroform extraction and ethanol precipitation. cDNA synthesis was performed either on 0.5 μg of total RNA (iScript Reverse transcription Supermix for RT-qPCR kit; Bio-Rad) or on 2 μg of total RNA for strand-specific reverse transcription (SuperScript II Reverse Transcriptase; Invitrogen or High-Capacity cDNA Reverse Transcription Kit; Appliedbiosystems). Gene-specific primers are available upon request. qRT-PCR analysis was performed on a Bio-Rad iCycler System (CFX) using SsoAdvanced SYBR Green Supermix (Bio-Rad). qRT-PCR conditions were 30 s at 95 °C, followed by 45 cycles of 5 s at 95 °C and 20 s at 63 °C. RT controls were performed by the omission of RNA template or RT enzyme. A specific standard curve was performed in parallel for each gene, and each sample was quantified in duplicate. Data were analyzed by gene regression using iCycler software and normalized to *Gapdh* or *36B4* levels.

### ChIP-seq

ChIP-seq data was previously generated^2^: ChIP-seq reads were aligned to the mouse reference genome (GRCm38/mm10) using Bowtie v0.12.8 using the following parameters -m 1 –strata –best. Only uniquely aligned reads have been retained for further analyses. Peak calling was performed using either MACS v1.4.2 or SICER v1.1^38^. MACS was used to detect peaks into RNAPII data using default parameters except for -g mm. SICER was used to detect islands into H3K27ac data using the script SICER.sh with the following parameters: Species: mm10, Threshold for redundancy allowed for chip reads: 1, Threshold for redundancy allowed for control reads: 1, Window size: 200 bps, Effective genome size as a fraction of the reference genome of mm10: 0.77, Gap size: 600 bps, Evalue for identification of candidate islands that exhibit clustering: 1000, False discovery rate controlling significance: 10^−2^. Fragment size was set according to the value assessed by Homer v4.7.2 makeTagDirectory. Peaks/Islands were annotated using Homer v4.7.2 with annotations extracted from Ensembl v78.

### RNAseq

RNA-seq data was previously generated^2^: RNAseq reads were aligned onto mouse rRNA sequences using bowtie^39^ release 0.12.7. Reads that do not map to rRNA sequences were mapped onto the mouse reference genome (GRCm38/mm10) using Tophat2 release 2.0.10^40^. Only uniquely aligned reads have been retained for further analyses.

Gene expression was quantified using HTSeq^41^ release 0.5.4p3 and gene annotations from Ensembl release 78. Read counts were normalized across libraries with the method proposed by Anders *et al*.^42^. Comparison between R6/1 and WT samples was performed using the method proposed by Love *et al*.^43^, implemented in the DESeq2 Bioconductor library (release 1.0.19). Adjustment for multiple testing was performed using a method previously described^44^.

### eRNA identification

In order to identify reads from putative eRNA, we removed split-mapped reads and reads that overlap (≥1 bp on the opposite strand) genes annotated by Ensembl (release 78) as: IG_C_gene, IGD_gene, IG_J_gene, IG_LV_gene, IG_V_pseudogene, Mt_rRNA, Mt_tRNA, polymorphic_pseudogene, protein_coding, pseudogene, rRNA, TR_V_gene and TR_V_pseudogene. We extended the region corresponding to those genes to 3 kb upstream of the transcription start site and 10 kb downstream of the transcript end site, in order to minimize signal from polymerase read-through from genic transcripts (as previously described ref. 19). IntersectBed from BEDTools release 2.21.0 was used for this purpose^45^. This overlap was performed on the opposite strand as the library preparation protocol we used to construct these RNAseq libraries leads to sequence the strand generated during first strand cDNA synthesis.

On those filtered RNAseq reads, we then detected the location of putative eRNA as genomic regions enriched in RNAseq reads. We thus used a method used for ChIP-seq peak detection: we used MACS v1.4.2^46^ with the following parameters –keep-dup = all –nomodel –nolambda -p 1e-4 –g mm. The parameter –shiftsize was set according to the value assessed by Homer v4.7.2^47^ makeTagDirectory.

#### Motif analysis

Motifs searching of known motifs (Jaspar 2014 motif database) was made using FIMO^48^ within sequences of up and down eRNA. FIMO results were then processed by a custom Perl script that computed the number of occurrence of each motif. To assess the enrichment of motifs within the regions of interest, the same analysis was done n times (n = 100) on randomly selected eRNA regions. We chose to use randomly selected eRNA regions as controls to compute an expected distribution of motif occurrence and to correct for the nucleotide composition bias that could occur specifically in eRNA sequences. Region size distribution of the randomly selected eRNA was the same as for the up and down eRNA of interest. The significance of the motif occurrence was estimated through the computation of a Z-score.

The Z-score was computed this way:


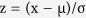


where:

−x is the observed value (number of motif occurrence)

−μ is the mean of the number of occurrences (computed on randomly selected data)

−σ is the standard deviation of the number of occurrences of motifs (computed on randomly selected data)

From the Z-scores, P values for each motif were computed. The P values were corrected for multi-testing using a method previously described^44^. Statistical analysis was done with custom R scripts.

#### Gene ontology (GO) analysis

Functional enrichments analyses were performed using the tools DAVID and/or GREAT^22^,^49^. For analyses with GREAT, defaults setting were used. Whole Mouse genome was used as background. Top-enriched terms are shown (*P* values < 0.05 were considered).

#### Integrated analyses

GalaxEast (galaxeast.fr) was used to integrate data relative to eRNA, mRNA, H3K27ac (including super-enhancers) and RNAPII signals.

#### Data access

RNA-seq and ChIP-seq data used for eRNA analysis are accessible through GEO (accession number GSE59572).

## Additional Information

**How to cite this article:** Le Gras, S. *et al*. Altered enhancer transcription underlies Huntington’s disease striatal transcriptional signature. *Sci. Rep.* **7**, 42875; doi: 10.1038/srep42875 (2017).

**Publisher's note:** Springer Nature remains neutral with regard to jurisdictional claims in published maps and institutional affiliations.

## Supplementary Material

Supplementary Figure S1

Supplementary Table S1

## References

[b1] Seredenina T. & Luthi-Carter R. What have we learned from gene expression profiles in Huntington’s disease? Neurobiol Dis 45, 83–98, doi: 10.1016/j.nbd.2011.07.001 (2012). 21820514

[b2] Achour M. . Neuronal identity genes regulated by super-enhancers are preferentially down-regulated in the striatum of Huntington’s disease mice. Hum Mol Genet 24, 3481–3496, doi: 10.1093/hmg/ddv099 (2015). 25784504

[b3] Valor L. M., Guiretti D., Lopez-Atalaya J. P. & Barco A. Genomic landscape of transcriptional and epigenetic dysregulation in early onset polyglutamine disease. J Neurosci 33, 10471–10482, doi: 10.1523/JNEUROSCI.0670-13.2013 (2013). 23785159 PMC6618595

[b4] Vashishtha M. . Targeting H3K4 trimethylation in Huntington disease. Proc Natl Acad Sci USA 110, E3027–3036, doi: 10.1073/pnas.1311323110 (2013). 23872847 PMC3740882

[b5] Seong I. S. . Huntingtin facilitates polycomb repressive complex 2. Hum Mol Genet 19, 573–583, doi: 10.1093/hmg/ddp524 (2010). 19933700 PMC2807366

[b6] Biagioli M. . Htt CAG repeat expansion confers pleiotropic gains of mutant huntingtin function in chromatin regulation. Hum Mol Genet, doi: 10.1093/hmg/ddv006 (2015). PMC4383859 25574027

[b7] Bai G. . Epigenetic dysregulation of hairy and enhancer of split 4 (HES4) is associated with striatal degeneration in postmortem Huntington brains. Hum Mol Genet 24, 1441–1456, doi: 10.1093/hmg/ddu561 (2015). 25480889 PMC4321450

[b8] Hodges A. . Regional and cellular gene expression changes in human Huntington’s disease brain. Hum Mol Genet 15, 965–977, doi: 10.1093/hmg/ddl013 (2006). 16467349

[b9] Kuhn A. . Mutant huntingtin’s effects on striatal gene expression in mice recapitulate changes observed in human Huntington’s disease brain and do not differ with mutant huntingtin length or wild-type huntingtin dosage. Hum Mol Genet 16, 1845–1861, doi: 10.1093/hmg/ddm133 (2007). 17519223

[b10] Langfelder P. . Integrated genomics and proteomics define huntingtin CAG length-dependent networks in mice. Nat Neurosci 19, 623–633, doi: 10.1038/nn.4256 (2016). 26900923 PMC5984042

[b11] Kuhn A. . Mutant huntingtin’s effects on striatal gene expression in mice recapitulate changes observed in human Huntington’s disease brain and do not differ with mutant huntingtin length or wild-type huntingtin dosage. Hum Mol Genet 16, 1845–1861 (2007). 17519223 10.1093/hmg/ddm133

[b12] Francelle L., Galvan L. & Brouillet E. Possible involvement of self-defense mechanisms in the preferential vulnerability of the striatum in Huntington’s disease. Front Cell Neurosci 8, 295, doi: 10.3389/fncel.2014.00295 (2014). 25309327 PMC4176035

[b13] Luthi-Carter R. . Decreased expression of striatal signaling genes in a mouse model of Huntington’s disease. Hum. Mol. Genet. 9, 1259–1271 (2000). 10814708 10.1093/hmg/9.9.1259

[b14] Valor L. M. Transcription, epigenetics and ameliorative strategies in Huntington’s Disease: a genome-wide perspective. Mol Neurobiol 51, 406–423, doi: 10.1007/s12035-014-8715-8 (2015). 24788684 PMC4309905

[b15] Wu H. . Tissue-specific RNA expression marks distant-acting developmental enhancers. PLoS Genet 10, e1004610, doi: 10.1371/journal.pgen.1004610 (2014). 25188404 PMC4154669

[b16] Kim T. K. . Widespread transcription at neuronal activity-regulated enhancers. Nature 465, 182–187, doi: 10.1038/nature09033 (2010). 20393465 PMC3020079

[b17] Malik A. N. . Genome-wide identification and characterization of functional neuronal activity-dependent enhancers. Nat Neurosci 17, 1330–1339, doi: 10.1038/nn.3808 (2014). 25195102 PMC4297619

[b18] Arner E. . Transcribed enhancers lead waves of coordinated transcription in transitioning mammalian cells. Science 347, 1010–1014, doi: 10.1126/science.1259418 (2015). 25678556 PMC4681433

[b19] Hah N. . Inflammation-sensitive super enhancers form domains of coordinately regulated enhancer RNAs. Proc Natl Acad Sci USA 112, E297–302, doi: 10.1073/pnas.1424028112 (2015). 25564661 PMC4311831

[b20] Schaukowitch K. . Enhancer RNA Facilitates NELF Release from Immediate Early Genes. Mol Cell 56, 29–42, doi: 10.1016/j.molcel.2014.08.023 (2014). 25263592 PMC4186258

[b21] Pnueli L., Rudnizky S., Yosefzon Y. & Melamed P. RNA transcribed from a distal enhancer is required for activating the chromatin at the promoter of the gonadotropin alpha-subunit gene. Proc Natl Acad Sci USA 112, 4369–4374, doi: 10.1073/pnas.1414841112 (2015). 25810254 PMC4394321

[b22] McLean C. Y. . GREAT improves functional interpretation of cis-regulatory regions. Nature biotechnology 28, 495–501, doi: 10.1038/nbt.1630 (2010). PMC4840234 20436461

[b23] Yuan J., Lei Z. N., Wang X., Deng Y. J. & Chen D. B. Interaction between Oc-1 and Lmx1a promotes ventral midbrain dopamine neural stem cells differentiation into dopamine neurons. Brain Res 1608, 40–50, doi: 10.1016/j.brainres.2015.02.046 (2015). 25747864

[b24] Ariano M. A. . Striatal potassium channel dysfunction in Huntington’s disease transgenic mice. J Neurophysiol 93, 2565–2574, doi: 10.1152/jn.00791.2004 (2005). 15625098

[b25] De Santa F. . A large fraction of extragenic RNA pol II transcription sites overlap enhancers. PLoS Biol 8, e1000384, doi: 10.1371/journal.pbio.1000384 (2010). 20485488 PMC2867938

[b26] Barkess G. & West A. G. Chromatin insulator elements: establishing barriers to set heterochromatin boundaries. Epigenomics 4, 67–80, doi: 10.2217/epi.11.112 (2012). 22332659

[b27] Bourillot P. Y. & Savatier P. Kruppel-like transcription factors and control of pluripotency. BMC Biol 8, 125, doi: 10.1186/1741-7007-8-125 (2010). 20875146 PMC2946285

[b28] Parisi S. . Direct targets of Klf5 transcription factor contribute to the maintenance of mouse embryonic stem cell undifferentiated state. BMC Biol 8, 128, doi: 10.1186/1741-7007-8-128 (2010). 20875108 PMC2955566

[b29] Ramanan N. . SRF mediates activity-induced gene expression and synaptic plasticity but not neuronal viability. Nat Neurosci 8, 759–767, doi: 10.1038/nn1462 (2005). 15880109

[b30] Cheng J. H., Pan D. Z., Tsai Z. T. & Tsai H. K. Genome-wide analysis of enhancer RNA in gene regulation across 12 mouse tissues. Sci Rep 5, 12648, doi: 10.1038/srep12648 (2015). 26219400 PMC4518263

[b31] Etkin A. . A role in learning for SRF: deletion in the adult forebrain disrupts LTD and the formation of an immediate memory of a novel context. Neuron 50, 127–143, doi: 10.1016/j.neuron.2006.03.013 (2006). 16600861

[b32] Cummings D. M. . Aberrant cortical synaptic plasticity and dopaminergic dysfunction in a mouse model of Huntington’s disease. Hum Mol Genet 15, 2856–2868, doi: 10.1093/hmg/ddl224 (2006). 16905556

[b33] Bargas J., Galarraga E. & Aceves J. Electrotonic properties of neostriatal neurons are modulated by extracellular potassium. Exp Brain Res 72, 390–398 (1988). 3224650 10.1007/BF00250260

[b34] Mermelstein P. G., Song W. J., Tkatch T., Yan Z. & Surmeier D. J. Inwardly rectifying potassium (IRK) currents are correlated with IRK subunit expression in rat nucleus accumbens medium spiny neurons. J Neurosci 18, 6650–6661 (1998). 9712637 10.1523/JNEUROSCI.18-17-06650.1998 PMC6792959

[b35] Guo J. U. . Neuronal activity modifies the DNA methylation landscape in the adult brain. Nat Neurosci 14, 1345–1351, doi: 10.1038/nn.2900 (2011). 21874013 PMC3183401

[b36] Liu H. . Systematic identification and annotation of human methylation marks based on bisulfite sequencing methylomes reveals distinct roles of cell type-specific hypomethylation in the regulation of cell identity genes. Nucleic Acids Res 44, 75–94, doi: 10.1093/nar/gkv1332 (2016). 26635396 PMC4705665

[b37] Mangiarini L. . Exon 1 of the *HD* gene with an expanded CAG repeat is sufficient to cause a progressive neurological phenotype in transgenic mice. Cell 87, 493–506 (1996). 8898202 10.1016/s0092-8674(00)81369-0

[b38] Zang C. . A clustering approach for identification of enriched domains from histone modification ChIP-Seq data. Bioinformatics 25, 1952–1958, doi: 10.1093/bioinformatics/btp340 (2009). 19505939 PMC2732366

[b39] Langmead B., Trapnell C., Pop M. & Salzberg S. L. Ultrafast and memory-efficient alignment of short DNA sequences to the human genome. Genome Biol 10, R25, doi: 10.1186/gb-2009-10-3-r25 (2009). 19261174 PMC2690996

[b40] Kim D. . TopHat2: accurate alignment of transcriptomes in the presence of insertions, deletions and gene fusions. Genome Biol 14, R36, doi: 10.1186/gb-2013-14-4-r36 (2013). 23618408 PMC4053844

[b41] Anders S. . Count-based differential expression analysis of RNA sequencing data using R and Bioconductor. Nat Protoc 8, 1765–1786, doi: 10.1038/nprot.2013.099 (2013). 23975260

[b42] Anders S. & Huber W. Differential expression analysis for sequence count data. Genome Biol 11, R106, doi: 10.1186/gb-2010-11-10-r106 (2010). 20979621 PMC3218662

[b43] Love M. I., Huber W. & Anders S. Moderated estimation of fold change and dispersion for RNA-seq data with DESeq2. Genome Biol 15, 550–571, doi: 10.1101/002832 (2014). 25516281 PMC4302049

[b44] Benjamini Y. & Hochberg Y. Controlling the False Discovery Rate - a Practical and Powerful Approach to Multiple Testing. J Roy Stat Soc B Met 57, 289–300 (1995).

[b45] Quinlan A. R. & Hall I. M. BEDTools: a flexible suite of utilities for comparing genomic features. Bioinformatics 26, 841–842, doi: 10.1093/bioinformatics/btq033 (2010). 20110278 PMC2832824

[b46] Zhang Y. . Model-based analysis of ChIP-Seq (MACS). Genome Biol 9, R137, doi: 10.1186/gb-2008-9-9-r137 (2008). 18798982 PMC2592715

[b47] Heinz S. . Simple combinations of lineage-determining transcription factors prime cis-regulatory elements required for macrophage and B cell identities. Mol Cell 38, 576–589, doi: 10.1016/j.molcel.2010.05.004 (2010). 20513432 PMC2898526

[b48] Grant C. E., Bailey T. L. & Noble W. S. FIMO: scanning for occurrences of a given motif. Bioinformatics 27, 1017–1018, doi: 10.1093/bioinformatics/btr064 (2011). 21330290 PMC3065696

[b49] Huang da W., Sherman B. T. & Lempicki R. A. Systematic and integrative analysis of large gene lists using DAVID bioinformatics resources. Nat Protoc 4, 44–57, doi: 10.1038/nprot.2008.211 (2009). 19131956

