# Supplementary material for: Altered enhancer transcription underlies Huntington’s disease striatal transcriptional signature
Source: Sci Rep. 2017 Feb 22;7:42875. doi: 10.1038/srep42875 (PMC5320509; doi:10.1038/srep42875)
Supplement: Supplementary Figure S1 [file srep42875-s1.pdf]

A

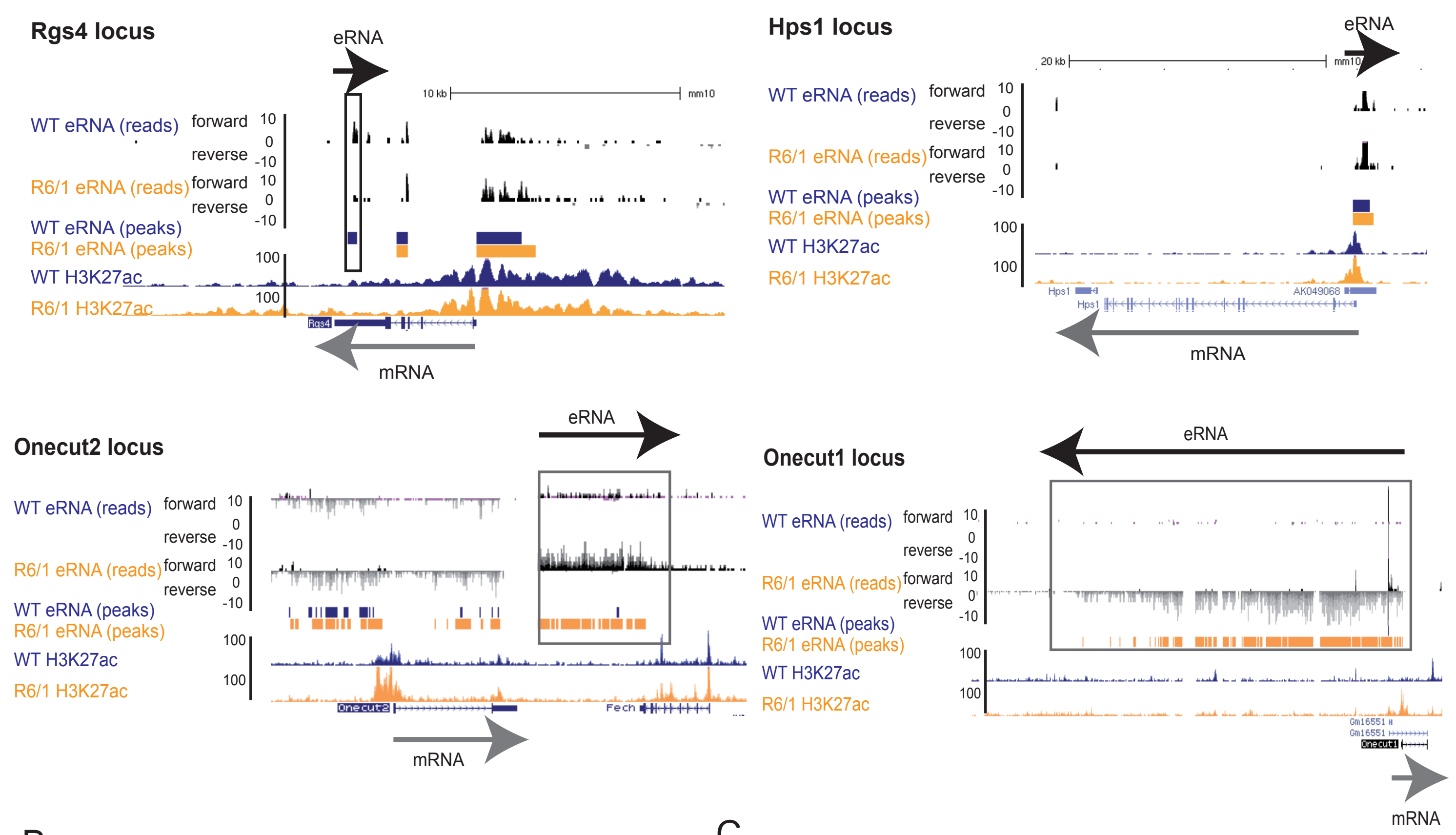

B

Gene name	R6/1 vs WT Log2 FC	R6/1 vs WT Adj pvalue	Q140 vs WT Log2 FC	Q140 vs WT Adj pvalue
Rgs4	-2,17	6E-63	-0,62	9E-33
Rgs9	-1,85	4E-73	-0,71	5E-29
Slc24a4	-1,76	1E-16	-0,23	3E-3
Chn1	-1,24	2E-75	-0,44	1E-20
Gpr6	-2,24	2E-108	-0,57	6-10
Ajap1	-0,99	9E-17	-0,23	2E-6
Bcr	-0,75	3E-11	-0,49	8E-21
Asphd2	-0,80	1E-8	-0,34	6E-9
Hps1	-0,26	2E-1	-0,05	5E-1

C

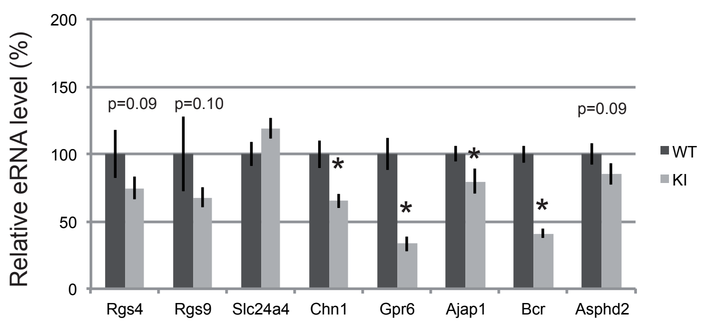

D

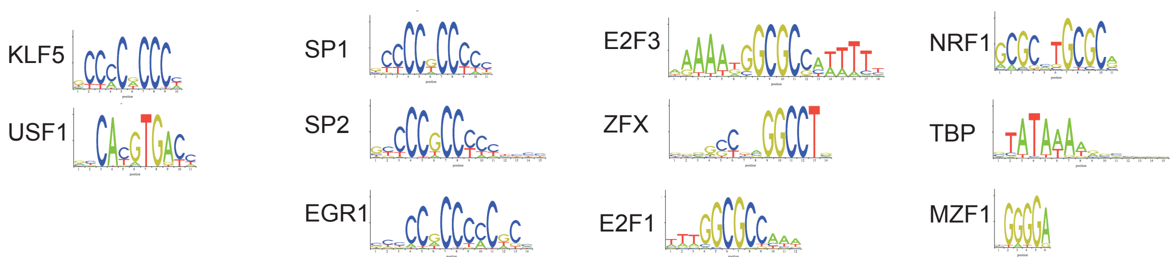

Altered enhancer transcription underlies Huntington's disease striatal transcriptional signature

Stéphanie Le Gras[1#], Céline Keime[1#], Anne Anthony[2,3#], Caroline Lotz[2,3], Lucie De Longprez[4,5], Emmanuel Brouillet[4,5], Jean-Christophe Cassel[2,3], Anne-Laurence Boutillier[2,3] and Karine Merienne[2,3*]

Figure S1. A. Genome browser representation of *Rgs4, Hsp1, Onecut1* and *Onecut2* loci, including eRNA reads (before H3K27ac filtering), eRNA peaks (after H3K27ac filtering) and H3K27ac signals in WT and R6/1 striatum. Black and grey arrows show the direction of expression of eRNA and mRNA, respectively. **B**. Table showing fold-changes (Log2 FC) and adjusted p-values (Adj pvalue) corresponding to differential expression of mRNAs transcribed from *Rgs4*, *Rgs9*, *Slc24a4*, *Chn1, Gpr6, Ajap1, Bcr* and *Asphd2*, analyzed from RNAseq data performed on 30 week-old R6/1 and WT striata (yellow box, Achour et al., 2015) and from RNAseq data performed on 10 month-old Q140 and control striata (green box, Langfelder et al., 2016). **C**. Levels of eRNAs associated with *Rgs4, Rgs9, Slc24a4, Chn1, Gpr6, Ajap1, Bcr* and *Asphd2* were measured from the striatum of 12 month-old Q140 mice using q-RT-PCR. Error bars, sem; *, $P < 0.05$ (Student's t-test). **D**. Sequences of DNA motifs enriched in enhancers leading to increased eRNAs in R6/1 vs WT striatum.

Table S1. List of differentially expressed eRNAs, including decreased and increased eRNAs. Adjusted p values and fold changes are <0.05 and > 1.25, respectively.
